# Comparison of biomechanical behavior between a cast material torso jacket and a polyethylene based jacket

**DOI:** 10.1186/1748-7161-10-S2-S15

**Published:** 2015-02-11

**Authors:** Robert Rizza, XueCheng Liu, John Thometz, Channing Tassone

**Affiliations:** 1 Department of Mechanical Engineering, Milwaukee School of Engineering Milwaukee, WI, USA; 2 Department of Orthopaedic Surgery, Medical College of Wisconsin, WI, USA

**Keywords:** TLSO, Cast, Early onset Scoliosis, brace design

## Abstract

**Background:**

Numerous designs are used to the treatment of Early Onset Scoliosis. For example, a Thoraco-Lumbo-Sacral Orthosis (TLSO) is constructed using Polyethylene (PE). In addition, a series of castings has been implemented using cast material (3M, BSN Medical). The cast material has some significant advantages over the PE design including: growth preserving, improved compliance, decreased invasiveness, delaying or avoiding surgery, and the ability to allow the skin to breathe. However, the mechanical effectiveness of the cast material brace as compared to the TLSO is unknown, thus providing the objective of this study.

**Methods:**

A total of 23 standardized tensile tests were performed on the Delta-Cast Soft^®^ and 3M^TM^ Scotchcast^TM^ Plus Casting Tape in order to obtain mechanical properties (Young’s and shear moduli and Poisson ratios). Using a radiograph of a thoracic spine, the size of twelve vertebrae and eleven intervertebral discs were measured and used to create a finite element spine model. Simulations using this model were used to establish mechanical loads which were then applied to finite element models of the TLSO and cast jacket. The thicknesses and number of material layers was varied in these models. Multiple simulations were performed.

**Results:**

It was found that a 6.6.mm thick cast jacket made of Delta-Cast Soft^®^ had a maximum deformation of 4.7 mm, a maximum stress of 2.9 MPa and a structural factor of safety of 5.71. On the other hand, a 4 mm thick jacket made of PE had a maximum deformation of 2 mm, a maximum stress of 8.9 MPa and a structural factor of safety of 2.70. The cast jacket was 3.5 times lighter and had a material of cost 1/5 of the PE brace.

**Conclusions:**

Based on the results, either design will generate the proper constraint forces to maintain spinal correction. But, based on the design parameters (thickness, mechanical properties, structural factor of safety and cost) the brace made of cast material, though slightly thicker has superior structural and cost benefits. Thus, from the biomechanical point of view, the cast brace is more efficient than the PE brace.

## Introduction

There are many designs in the marketplace for the treatment of Early Onset Scoliosis. One design in particular the Thoraco-Lumbo-Sacral Orthosis (TLSO-Wilmington, Boston etc.) makes use of High Density Polyethylene (HDPE). Similar designs are also possible with Low Density Polyethylene (LDPE). Nevertheless, these designs employ a polymer material and are padded with a foam material (usually Urethane based).

Another design employs a series of castings without any foam padding. This design uses a cast tape which has several distinct advantages compared to PE. These advantages include: growth preserving, improved compliance, decreased invasiveness, delaying or avoiding surgery, and the ability to allow the skin to breathe. Furthermore, the cast material is made of a biodegradable water based resin with less environmental impact compared to PE [1].

**Figure 1:**
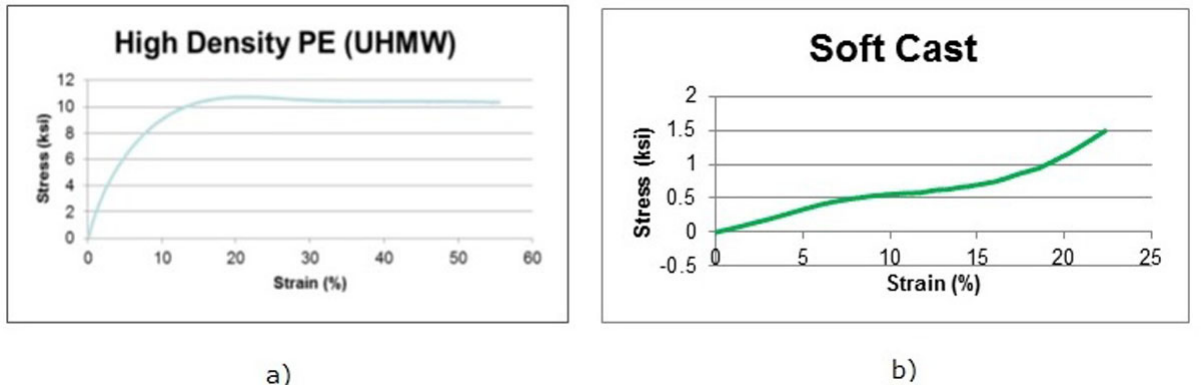
**a) Stress-Strain curve for HDPE and b) for cast material**. (The figure is not original and the original can be found in the abstract published in the Standard Supplement of the Sapporo 2014 IRSSD meeting, [6]).

The material behavior of the PE and cast material is quite different because the materials are significantly different structurally. These structural differences lead to stress-strain curves that show completely dissimilar behavior (Figure 1). In fact, the PE behaves as a ductile material with a long perfectly plastic region whereas the cast material is hyper-elastic.

**Figure 2:**
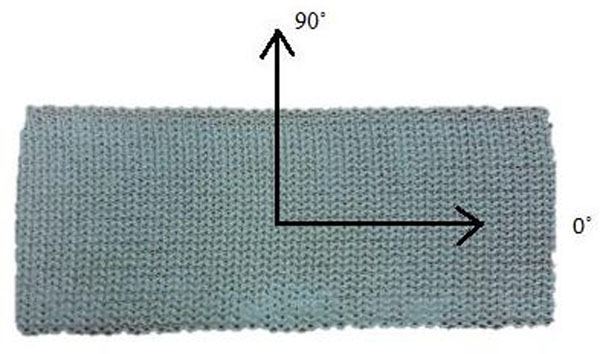
**Fiber Orientation of the Cast Materials.** (The figure is not original and the original can be found in the abstract published in the Standard Supplement of the Sapporo 2014 IRSSD meeting, [6]).

Structurally, cast materials are bi-woven and have reinforcing fibers oriented in both the 0º (“1”) and 90º (“2”) direction. The 0º direction fibers run the length of the cast material and the 90º direction fibers run along the width as shown in Figure 2. Thus, this material may be modeled as an Orthotropic material with 4 independent material constants (E_11_, E_22_, G_12_, and ν_12_) [2].

The mechanical effectiveness of a cast material brace compared to a PE brace is unknown. Thus the objective of this study was to compare the cast and PE designs mechanically. Furthermore, there is a lack of information regarding mechanical properties of cast material and the lack of a suitable finite element model of the cast brace. Thus, additional objectives of this study were to obtain mechanical properties of the cast material and develop such a suitable model.

## Materials and methods

Material testing was performed on the Delta-Cast Soft^®^ (BSN Medical, Hamburg) and 3M^TM^ Scotchcast^TM^ Plus Casting Tape (3M Health Care Limited, Loughborough) in order to obtain the Young (E_11_, E_22_), shear (G_12_) moduli, and Poisson ratio (ν_12_). A total of 23 specimens (0.5 in wide x 8 in long) were tested to failure (in the 0º, 90º and 45º direction) using an MTS Alliance RT/50 (MTS, Eden Prairie, MN).

Using a radiograph of a patient’s thoracic spine, the size of twelve vertebrae and eleven intervertebral discs were measured and used to create a spine model based on [3] with SolidWorks (Dassault Systèmes, Waltham, MA) The spine model was imported into ANSYS (ANSYS, Canonsburg, PA). To mimic, in vivo behavior, T12 was completely fixed and T1 was only allowed to displace vertically. A 3 N/mm load was applied in the sagittal plane to the T7, T8 and T9 vertebrae to generate a 13° reduction in Cobb angle. Linear mechanical properties were used for the vertebrae (E=10 GPa, ν=0.3) and disc (E=4.2 MPa , ν=0.45) [4]. Results of the spine model were used to calculate appropriate mechanical loading needed to correct the spinal deformity [5] and applied to the Torso Jacket model.

**Figure 3:**
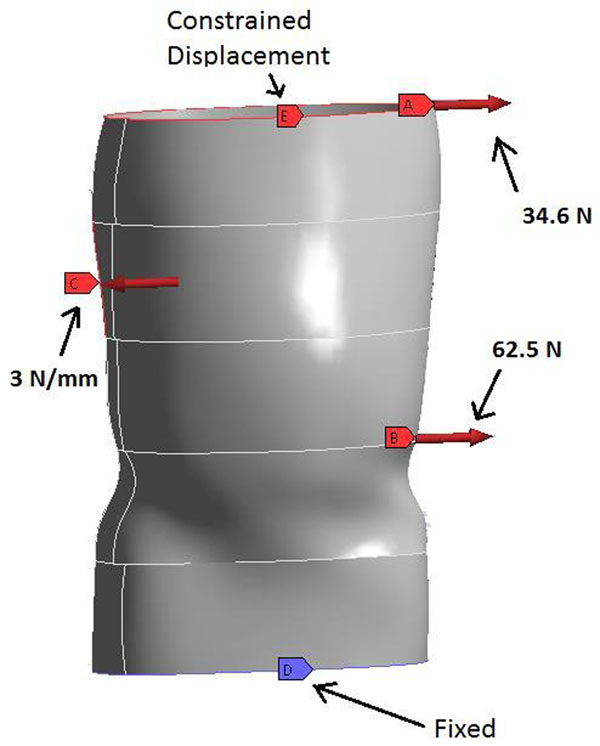
Finite Element model of a scanned Torso Jacket.

A Torso Jacket model was constructed using SolidWorks and scanned geometry of a patient’s brace (BMI, New Berlin, WI) and imported into ANSYS (Figure 3). Based on the spine Model, restraint forces of 34.6 N and 62.5 N were applied at T1 and T12 levels. Also a 3 N/mm load was applied corresponding to the T7, T8 and T9 vertebrae. The spine model predicted a maximum allowable brace deformation of 4.9 mm, otherwise the brace would not apply the necessary reaction forces to main spinal correction. Thus, this deformation was used as a design criterion for all the braces. Simulations were carried out with the cast and PE material (E = 130 ksi and v= 0.46) for varying thicknesses.

## Results

The Young’s and shear moduli as well as Poisson ratio for the cast materials are given in Table 1. We see that the 3M^TM^ Scotchcast^TM^ Plus Casting Tape is stiffer in the 0° direction than the Delta-Cast Soft^®^ tape (26.7 ksi versus 16.1 ksi). The other properties are similar.

**Table 1:** Comparison of mechanical properties between Delta-Cast Soft^®^ and 3M^TM^ Scotchcast^TM^ Plus Casting Tape.

Property	Delta-Cast Soft^®^	3M^TM^ Scotchcast^TM^ Plus
E_11_	16.6 ksi	26.7 ksi

E_22_	10.5 ksi	11.9 ksi

G_12_	1.7 ksi	1.1 ksi

ν_12_	0.3035	0.3115

**Figure 4:**
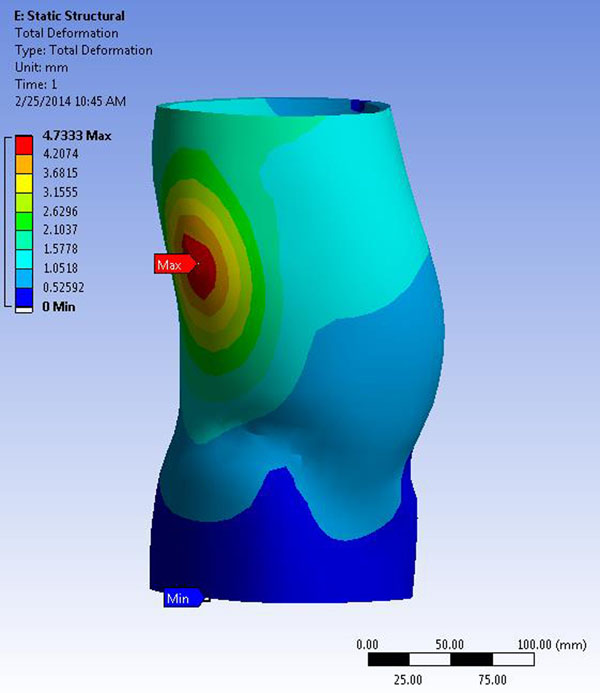
Finite Element results of the jacket for 6 layers of Delta-Cast Soft^®^.

In Figure 4, we see typical results of the finite element analysis of the Torso Jacket model. In this case, results are shown for 6 layers of Delta-Cast Soft^®^. The results indicate a maximum deformation of 4.7 mm on the lateral side of the brace.

The thickness, factor of safety, maximum deformation and maximum stress for each simulated brace can be seen in Table 2. The material cost of each design is given in Table 3. We see that six layers of Delta-Cast Soft^®^ and the HDPE show a maximum deformation that is lower than the allowable deformation of 4.9 mm. Six layers of Delta-Cast Soft^®^ has a factor of safety twice as high as the HDPE and costs about fifteen dollars less.

**Table 2:** Maximum stress, structural factor of safety and maximum deformation for the various brace designs.

Material/Schedule	Thickness (mm)	Maximum Stress [MPa]	Factor of Safety	Maximum Deformation [mm]
Cast Soft^®^/3 Layers	3.3	8.7	1.9	17.7

Cast Soft^®^/4 Layers	4.4	5.4	3.06	10.2

Cast Soft^®^/5 Layers	5.5	3.8	4.35	6.4

Cast Soft^®^/6 Layers	6.6	2.9	5.71	4.7

LDPE/3 Layers	4	8.9	1.12	3.9

HDPE/3 Layers	4	8.9	2.7	2

**Table 3:** Cost of each brace design

Material/Schedule	Cost per m^2^ (US Dollars)	Total Material Cost (US Dollars)
Cast Soft^®^/3 Layers	8.65	25.95
		
Cast Soft^®^/4 Layers		34.6
		
Cast Soft^®^/5 Layers		43.25
		
Cast Soft^®^/6 Layers		51.9

LDPE/3 Layers	35.73	66.6

HDPE/3 Layers	22.2	66.6

## Conclusions

In the study, the mechanical properties of cast material were found. There is some difference between the manufactures primarily in the 0° direction, which provides the majority of the brace’s circumferential stiffness and strength. It must be noted that he properties of the cast material are orthotropic as suggested by the cellular type structure of the cast tape.

In comparing the cast brace design to the PE based design, the cast material with the lesser mechanical properties was used as this is the worst case design scenario.

The results of the study indicate that it is indeed possible to design a brace of cast material for the treatment of Early Onset Scoliosis that will be mechanically compatible to a TLSO brace made of PE. This is true because both designs will be able to generate the mechanical loads necessary for spinal correction.

However, as can be seen by examining Tables 2 and 3, the brace made of cast material is slightly thicker (6 mm compared to 4 mm) but costs about 1/5 less and has double the strength of the PE brace. Furthermore, because the cast material is significantly less dense than PE (0.173 g/cm^3^ versus 0.924 g/cm^3^) the cast brace is 3.5 times lighter. Thus, the cast brace design is more efficient than the HDPE design.

This is the extended abstract of IRSSD 2014 program book [6].

## Limitations

In the spine model, only curvature in the Coronal plane was considered. Furthermore, only 1 radiograph from 1 patient was used to determine the equivalent mechanical loads applied to the Torso Jacket Model. In addition, the brace designs were not compared in-vivo.

## Nomenclature

E, E_11_, E_22_……Young’s moduli

G_12_….Shear modulus

ν, ν_12_…..Poisson ratio

HDPE…High Density Polyethylene

LDPE….Low Density Polyethylene

Orthotropic material…..Material having has two or three mutually orthogonal axes of rotational symmetry

Tensile test…..A mechanical test performed by pulling a specimen in tension to failure

## Competing interests

The authors declare that they have no competing interests.

## Authors’ contributions

RR performed the material testing and FEA analysis data collection and analysis, and helped to draft the manuscript. All authors designed and coordinated the study. JT and CT provided anatomical data of the torso. XL and RR performed data analysis, and drafted the manuscript. All authors read and approved the final manuscript.

## Declarations

Publication charges for this collection were funded by IRSSD 2014 Sapporo.

This article has been published as part of *Scoliosis* Volume 10 Supplement 2, 2015: Research into Spinal Deformities 9: Short Papers from the IRSSD 2014 Meeting. The full contents of the supplement are available online at http://www.scoliosisjournal.com/supplements/10/S2.
